# Cytokine-induced cysteine- serine-rich nuclear protein-1 (CSRNP1) selectively contributes to *MMP1* expression in human chondrocytes

**DOI:** 10.1371/journal.pone.0207240

**Published:** 2018-11-15

**Authors:** Christopher D. Macdonald, Adrian M. D. Falconer, Chun Ming Chan, David J. Wilkinson, Andrew Skelton, Louise Reynard, Gary J. Litherland, G. Nicholas Europe-Finner, Andrew D. Rowan

**Affiliations:** Skeletal Research Group, Institute of Genetic Medicine, Newcastle University, Newcastle-upon-Tyne, United Kingdom; Augusta University, UNITED STATES

## Abstract

Irreversible cartilage collagen breakdown by the collagenolytic matrix metalloproteinases (MMPs)-1 and MMP-13 represents a key event in pathologies associated with tissue destruction such as arthritis. Inflammation is closely associated with such pathology and occurs in both rheumatoid and osteoarthritis making it highly relevant to the prevailing tissue damage that characterises these diseases. The inflammation-induced activating protein-1 (AP-1) transcription factor is an important regulator of both *MMP1* and *MMP13* genes with interplay between signalling pathways contributing to their expression. Here, we have examined the regulation of *MMP1* expression, and using *in vivo* chromatin immunoprecipitation analyses we have demonstrated that cFos bound to the AP-1 *cis* element within the proximal *MMP1* promoter only when the gene was transcriptionally silent as previously observed for *MMP13*. Subsequent small interfering RNA-mediated silencing confirmed however, that cFos significantly contributes to *MMP1* expression. In contrast, silencing of *ATF3* (a prime *MMP13* modulator) did not affect *MMP1* expression whilst silencing of the Wnt-associated regulator cysteine- serine-rich nuclear protein-1 (*CSRNP1*) resulted in substantial repression of *MMP1* but not *MMP13*. Furthermore, following an early transient peak in expression of CSRNP1 at the mRNA and protein levels similar to that seen for cFOS, *CSRNP1* expression subsequently persisted unlike cFOS. Finally, DNA binding assays indicated that the binding of CSRNP1 to the AP-1 consensus-like sequences within the proximal promoter regions of *MMP1* and *MMP13* was preferentially selective for *MMP1* whilst activating transcription factor 3 (ATF3) binding was exclusive to *MMP13*. These data further extend our understanding of the previously reported differential regulation of these MMP genes, and strongly indicate that although cFos modulates the expression of *MMP1/13*, downstream factors such as CSRNP1 and ATF3 ultimately serve as transcriptional regulators in the context of an inflammatory stimulus for these potent collagenolytic MMPs.

## Introduction

Degenerative joint diseases result in destruction of the articular cartilage, typically when a variety of pro-inflammatory mediators perpetuate the disease process (reviewed in [1]). There is now increasing evidence that such inflammatory mediators are involved the pathogenesis of both rheumatoid arthritis (RA) and osteoarthritis (OA) [1]. These mediators include interleukin (IL)-1, members of the IL-6 family (including IL-6 and oncostatin M (OSM)) as well as other inducers of signalling such as Wnt [2–4]. Cartilage collagen breakdown is well documented to be primarily due to the matrix metalloproteinases (MMPs), in particular the collagenases MMP-1 and MMP-13, which are responsible for the irreversible degradation of the collagenous matrix [5]. Although dogma suggests MMP-1 is the major collagenase in RA, whilst MMP-13 prevails in OA, the above mediators all drive the expression of both MMPs with *MMP1* induction typically more marked [6, 7]. Furthermore, we have previously reported differential regulatory mechanisms for cytokine-induced *MMP1* and *MMP13* expression in human chondrocytes [8, 9], and recently confirmed that the most potent cytokine stimulus reported promotes many signalling pathways that are common to a wide variety of pro-inflammatory stimuli [10]. Indeed, we and others have demonstrated marked *MMP* expression in chondrocytes stimulated with numerous inflammatory mediators [11–15] indicating such potent expression occurs within most inflammatory situations associated with tissue destruction such as RA and OA. Consequently, the collagenases represent key therapeutic targets for disease-modifying agents [5, 16].

Investigations of the molecular mechanisms by which MMPs are regulated [17, 18] have highlighted a primary role for activator protein (AP)-1 transcription factor complexes in regulating MMP expression [9, 19–21]. AP-1 exists as a protein dimer found ubiquitously throughout different tissues and cell types and is comprised of FOS and JUN family heterodimers. Many studies [13, 22–24], including our own [9, 17, 25], have indicated that cFos/cJun AP-1 heterodimers act as critical regulators of *MMP1* and *MMP13* in chondrocytes following pro-catabolic stimuli such as IL-1+OSM. Interestingly, despite the importance of cFos (gene name *FOS*), differential regulation of *MMP1* and *MMP13* via specific cell signalling pathways [8, 9, 13–15, 24, 26] suggests more complex mechanism(s) underpin the transcriptional activation of these MMPs. In this context, we have recently shown that *MMP13* expression is initially modulated by a transient expression of cFos, which subsequently results in the prolonged expression of activating transcription factor 3 (ATF3) to promote *MMP13* expression via an AP-1 cis element within the *MMP13* proximal promoter [10].

In order to expand our understanding of *MMP1* regulatory mechanisms, *MMP1* regulation was assessed following the same potent pro-catabolic stimulus as detailed above for *MMP13*. Data herein indeed confirm that cFos silencing was sufficient to significantly inhibit *MMP1* expression; however, *ATF3* silencing did not affect *MMP1* expression. Importantly, IL-1+OSM stimulation induced cysteine- serine-rich nuclear protein-1 (CSRNP1; also termed Axin upregulated-1 (AXUD1)), expression to selectively modulate *MMP1* in human chondrocytes. CSRNP1 was first identified as being induced by the β-catenin regulator, AXIN1[27], and has subsequently been characterised as a transcription factor having a role in the regulation of Wnt signalling[28]. In this respect the Wnt/β-catenin signalling cascade pathway has recently been shown to play an important regulator in the onset and progression of OA[29].

This is the first report indicating a role for CSRNP1 in modulating the expression of a chondrogenic collagenase due to an inflammatory insult, and may be indicative of diverse regulatory moieties affecting specific *MMP* gene transcription as a consequence of inflammation associated with OA progression.

## Materials and methods

### Materials

All chemicals were of the highest purity available and obtained from Sigma Chemical Co (Poole, UK) unless otherwise stated. All cytokines were recombinant human. IL-1α was a generous gift from Dr Keith Ray (GlaxoSmithKline, Stevenage, UK). OSM was prepared in-house as described [30]. siRNA reagents were screened for toxicity using the Toxilight assay of adenylate kinase release (Lonza, Wokingham, UK).

### Chondrocytes

Human chondrocytes were isolated by enzymatic digestion of macroscopically normal articular cartilage from OA patients undergoing joint replacement surgery as described [31]. All subjects gave informed consent and the study was approved by the Newcastle and North Tyneside Joint Ethics Committee. Chondrocytes were maintained in Dulbecco’s modified Eagle’s medium supplemented with 10% foetal bovine serum, 100 IU/mL penicillin, 100 μg/mL streptomycin, 40 U/mL nystatin.

### Gene expression analyses

RNA was typically stabilised in cell lysates in a 96-well format and cDNA synthesised using the Cells-to-Signal kit (Life Technologies) as directed. SYBR Green real-time PCR or Taqman assays (using Universal Probe Library probes (Roche Applied Sciences)), referred to herein as qPCR, were conducted using primers and conditions described previously [8–10] except for *MMP1*: For, 5’-CGAATGGCTCATTAAATCAGTTATGG-3’ and Rev, 5’-TATTAGCTCTAGAATTACCACAGTTATCC-3’, Probe, FAM-CAGAGAGTACAACTTACA TCGTGTTGCGGCTC-TAMRA; *MMP1* (proximal promoter/transcription start site): For, 5’-TCAGTACAGGTGCCGAACAG-3’ and Rev, 5’-CAAGATGTGTGCGAAGGAGA-3; *MMP1* (3’ untranslated region): For, 5’-ACTGAATGGGCAAAAACTGG-3’ and Rev, 5’-TGCAAACAGGGACAATTTGA-3’; *CSRNP1*: For, 5’-CCTGCCTGACCGTGACTT-3’ and Rev, 5’-AGCCCGCTTCAGGATAGAC-3’, Probe: 57. Nascent hnRNA transcript expression of *MMP1* was determined via reverse transcription and Taqman employing 5′-TTTCTTGATTGGCAGGGAA-3′ (For) and 5′-GGAAGCCAAAGGAGCTGTA-3′ (Rev) primers spanning the Exon8/Intron7 boundary with probe number 26.

### Chromatin immunoprecipitation (ChIP)

ChIP experiments were performed according to the standard protocol detailed in the EZ-ChIP kit (Merck-Millipore). Briefly, human chondrocytes were cultured until 70–80% confluent at which point they were treated with IL-1 (0.05 ng/mL) in combination with OSM (10 ng/mL) for various durations. Cells were then subject to cross-linking with formaldehyde for 5 min with agitation, and the reaction quenched with 0.125 M glycine for 5 min (with agitation). Cells were then washed twice and scraped into PBS supplemented with protease inhibitors. Cells were pelleted, resuspended in lysis buffer and lysates sonicated in a Bioruptor Plus (Diagenode, Liege, Belgium) sonicating water bath (15 cycles of 30 sec on, 30 sec off at full power) to shear the chromatin. Samples were then pre-cleared with the appropriate agarose beads for 1 h and subject to an overnight immunoprecipitation with relevant antibodies (10 μg) or corresponding isotype controls; anti-phospho-RNA polymerase II CTD repeats YSPTSPS (Ser5; pRNA Pol II) was from Abcam (Cambridge, UK); anti-acetyl(Lys^9/14^)-histone H3 (AcH3; cat no. 06–599) was from Millipore (Dundee, UK). Antibody/protein/DNA complexes were extracted with agarose beads which were washed sequentially with kit-supplied buffers. DNA was then eluted and crosslinks reversed (4 h incubation at 65 °C with 5 M NaCl, and then 1 h incubation at 37 °C with RNase (1 IU) and proteinase K (1 IU) to degrade RNA and protein, respectively). DNA was purified using spin columns and then used as input for SYBR green qPCR using primers outlined above. Data were normalised to background using isotype control antibodies and variations in amount of genomic DNA were normalised using Input.

### Gene promoter profiling

*In silico* analyses on ChIPSeq (cJun) MMP1/13 data [32] to identify the presence of putative CSRNP1 binding sites (AGAGTN [33]) were performed using ENCODE [34].

### Cell fractionation and immunoblotting

Chondrocyte lysates were prepared as described previously [8, 31]. In some experiments chondrocytes were subjected to subcellular fractionation using NE-PER Nuclear and Cytoplasmic Protein Extraction Kit or Subcellular Protein Fractionation Kit (both from ThermoFisher Scientific, Loughborough, UK). Lysates or fractions were resolved by SDS-PAGE, transferred to PVDF membranes and subsequently probed with the following antibodies: ATF3 (sc188) and CSRNP1 (sc81191) (used on cellular fractionation and experiments utilising nuclear lysates) purchased from Santa Cruz Biotechnology (Santa Cruz, CA); and CSRNP1 (ab103708) (used on whole cell lysates) from Abcam (Cambridge, UK). The specificity of all antibodies was confirmed using chondrocyte lysates (see full-length blots in S1 Fig), whilst blots were cropped for clarity of comparison.

### Cell transfection and RNA interference

Primary human chondrocytes were prepared and cultured as above, and transfected as described previously [8]. Dharmacon ON-TARGET plus SMARTpool (ThermoFisher Scientific, Loughborough, UK) of 4 specific siRNA duplexes (100 nM siRNA final concentration) were used throughout to assess IL-1+OSM-induced *MMP1* expression. All siRNAs used were as previously described [10] except for *CSRNP1* (Cat. L-013653; Dharmacon), BMP2 (Cat. L-011219; Dharmacon), IER2 (Cat. L-019884; Dharmacon) and IER5 (Cat. L-013467; Dharmacon). Following transfection, cells were stimulated for 1.25 h to measure transcriptional regulators, or 24 h for *MMP1*. Depletion of gene-specific mRNA levels was calculated by comparison of expression levels with cells transfected with 100 nM siCONTROL (siCon: non-targeting siRNA #2, Cat. 001210–02; Dharmacon).

### DNA affinity precipitation assays (DAPA)

Chondrocyte nuclear lysates were generated as described above. Nuclear protein (100–300 μg) was incubated with double stranded biotinylated oligonucleotides (45 pmol) containing either the proximal AP-1 binding site for *MMP1* (5’-[biotin]-TAAAGCATGAGTCAGACACCT-3’; 5’-AGGTGTCTGACTCATGCTTTA-3’) or *MMP13* (5’-[biotin]-TAAGTGATGACTCACCATTGC-3’; 5’-GCAATGGTGAGTCATCCTTA-3’) in 500 μl of binding buffer (12 mM HEPES pH 7.9, 4 mM Tris-HCl, 60 mM KCl, 5% (w/v) glycerol, 0.5 mM EDTA, 1 mM DTT and 1 mini protease inhibitor cocktail tablet/10 mL of buffer) for 1 h at 4 °C with rotation. Streptavidin-coated magnetic dynabeads (ThermoFisher Scientific) were then added and incubated for 2 h with rotation at 4 °C. Dynabead-bound protein/DNA complexes were isolated using a Dynabead magnet (ThermoFisher Scientific). Beads were resuspended in 50 μl of SDS-PAGE sample buffer, incubated at 100 °C for 5 min, pelleted and supernatants removed for immunoblotting (as above) except that each individual blot was cut to provide two blots with proteins of molecular mass ≥35kDa or ≤35kDa for CSRNP1 and ATF3 probing, respectively. Specificity of binding was determined in the presence of 50x excess non-biotinylated AP-1 oligonucleotides.

### Statistical analyses

Statistical differences between sample groups were assessed using one-way analysis of variance (ANOVA) with a post-hoc Bonferroni’s multiple comparison test or Student’s 2-tailed unpaired *t*-test, where ***p<0.001, **p<0.01, *p<0.05. For clarity, only selected comparisons are presented in some figures.

## Results

### *MMP1* gene expression is temporally delayed in cytokine-stimulated chondrocytes

**Fig 1:**
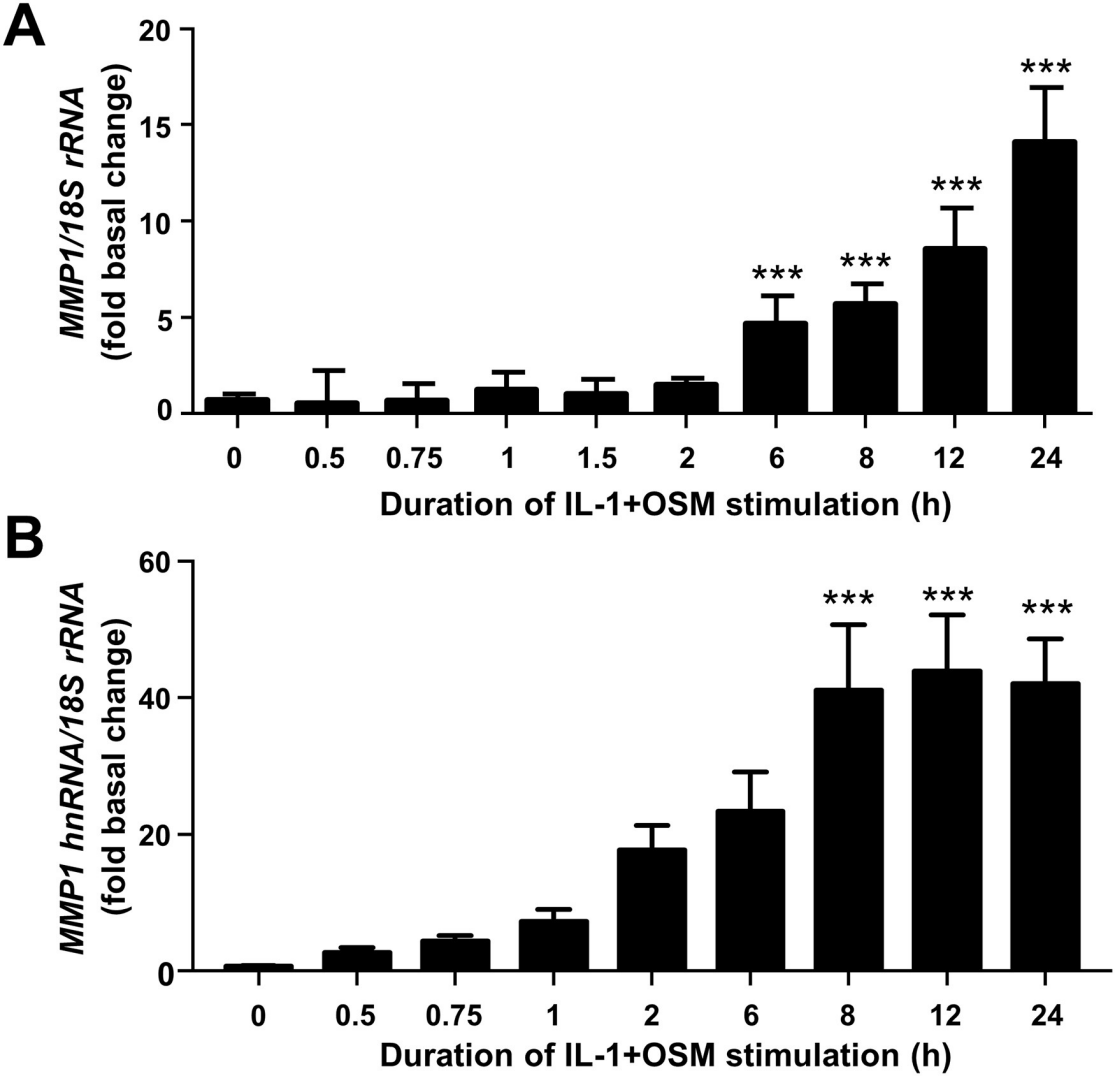
IL-1+OSM-induced *MMP1* mRNA expression is preceded by nascent hnRNA formation. Chondrocytes were stimulated with IL-1 (0.05 ng/mL) in combination with OSM (10 ng/mL) for the indicated durations. Total RNA was isolated, reverse transcribed and subjected to qPCR for (A) *MMP1* mRNA or (B) *MMP1* nascent hnRNA as described in the Materials and Methods. Data are expressed relative to 18S rRNA and presented as fold increase compared to basal expression. qPCR data (n = 4) are representative of at least three separate chondrocyte populations. All data are presented as mean (± SD), where ***, p<0.001; **, p<0.01; *, p<0.05 IL-1+OSM-treated compared to control; ANOVA.

**Fig 2:**
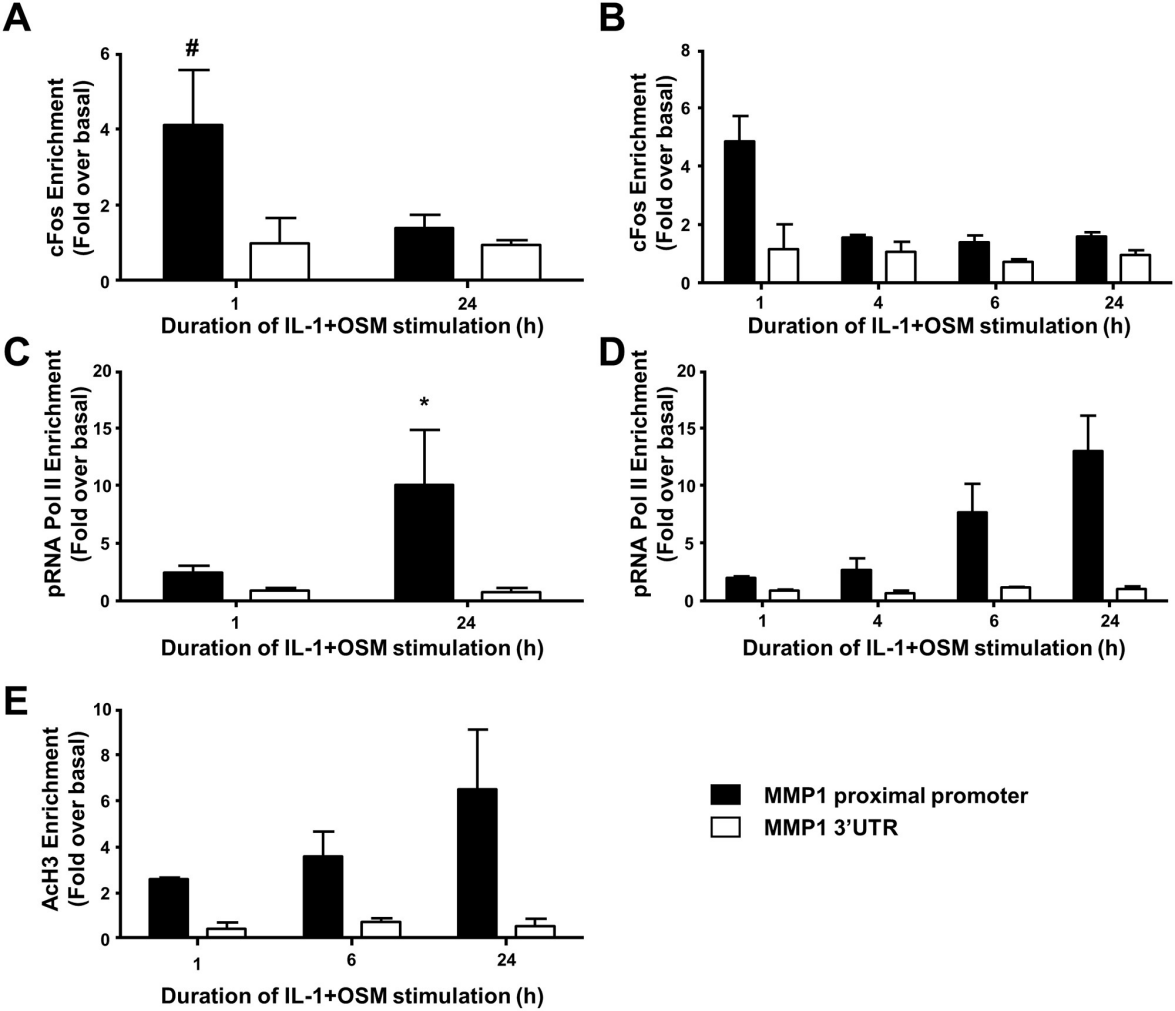
ChIP analyses of the *MMP1* proximal promoter. Human chondrocytes were treated with IL-1 (0.05 ng/mL) in combination with OSM (10 ng/mL) for the indicated durations. Cells were then subject to DNA-protein cross-linking, lysis and DNA shearing. Immuno-precipitation for cFOS (A,B), pRNA Pol II (C,D) or AcH3 (E) was followed by isolation of complexed genomic DNA and qPCR for the proximal (closed bars) and 3’-UTR (used for normalization; open bars) regions of *MMP1* as indicated. Data (mean ± SD, n = 4) are pooled from at least three separate chondrocyte populations from different donors. Statistical comparisons are: ^#^, p<0.05 (1 h IL-1+OSM stimulation versus basal); *, p<0.05 (24 h IL-1+OSM stimulation versus basal).

**Fig 3:**
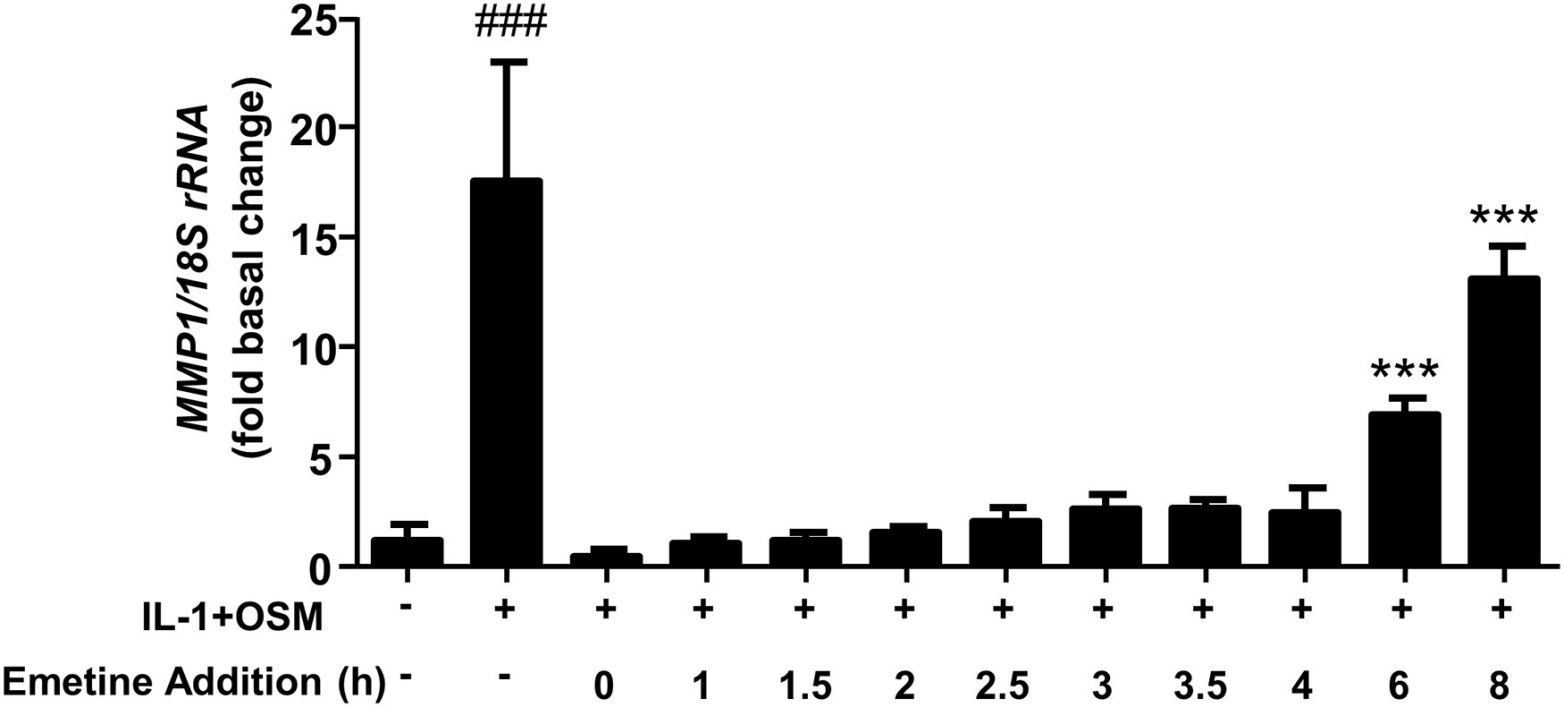
IL-1+OSM-induced *MMP1* expression is dependent on new protein synthesis. Chondrocytes were treated with IL-1 (0.05 ng/mL) and OSM (10 ng/mL) for 24 h. Emetine (10 μM final concentration) was added at the indicated times after initiation of IL-1+OSM stimulation and qPCR performed on extracted RNA. Relative expression levels of *MMP1* mRNA were normalized to 18S rRNA, where ^###^, p<0.001 (IL-1+OSM vs basal); ***, p<0.001 (IL-1+OSM+emetine vs basal). Data (mean ± SD, n = 6) are representative of three separate experiments each using chondrocyte cultures from different donors.

We have previously shown that the potent cytokine combination of IL-1+OSM demonstrates a time-dependency for collagenolytic MMP induction, typically reaching maximal induction at 24 h [6, 7, 35]. This dependency is confirmed here where primary human articular chondrocytes expressed *MMP1* mRNA within 6 h post-stimulation (Fig 1A) in line with previous findings [7]. Importantly, nascent hnRNA mirrored the observed mRNA increase (Fig 1B) indicating *MMP1* transcription occurred independently of mRNA synthesis/stability. Furthermore, previous data indicate that c*FOS* is rapidly, but transiently, expressed following IL-1+OSM stimulation [10, 25]; employing ChIP assays herein cFos was observed to be enriched at the proximal AP-1 element of the *MMP1* promoter at 1 h post-stimulation (Fig 2A and 2B). However, ChIP assays also confirmed enhanced enrichment of phosphorylated RNA Pol II to the proximal *MMP1* promoter 24 h post-stimulation, but not at 1 h (Fig 2C and 2D), whilst the time-dependent recruitment of acetylated histone H3 to the *MMP1* promoter (Fig 2E) reflected the observed time course of *MMP1* mRNA expression. Our previous data indicated a requirement for new protein synthesis for *MMP13* induction [10] by employing the protein synthesis inhibitor emetine. Similarly, in this study chondrocytes were stimulated for 24 h with IL-1+OSM and emetine added subsequently for varying durations throughout the stimulation which significantly reduced cytokine-induced *MMP1* expression and persisted for at least 6–8 h post-stimulation (Fig 3).

**Fig 4:**
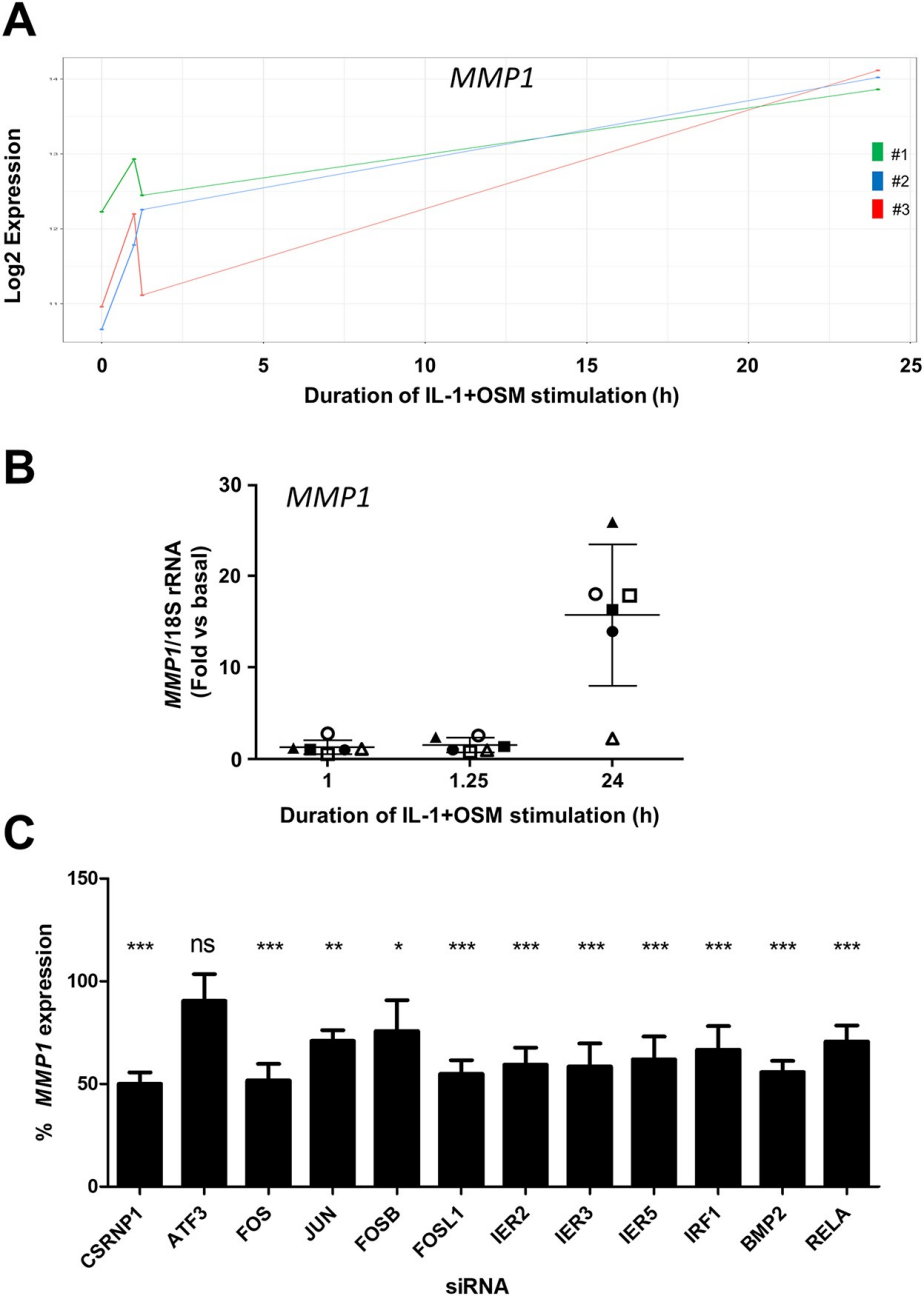
IL-1+OSM-induced *MMP1* expression is maximal at 24 h post-stimulation. Human chondrocytes were treated with IL-1 (0.05 ng/mL) in combination with OSM (10 ng/mL) for the indicated time points and total RNA isolated. (A) RNA from three separate populations (#1, #2 and #3) was profiled using the Human HT-12v4 Expression Beadchip. The expression profile for *MMP1* from the microarray dataset for each chondrocyte population is shown. (B) RNA from six separate chondrocyte populations was subjected to qPCR for *MMP1*, and relative expression levels (normalized to 18S rRNA) at the indicated times determined. The plots show mean ± SD (n = 6). Data from the populations used in the microarray analyses are highlighted by the solid symbols (#1, ▲; #2 ■; #3, ●). (C) Prior to stimulation with IL-1 (0.05 ng/ml) in combination with OSM (10 ng/ml), human chondrocytes were transfected with siRNA specific for the indicated genes, or a non-targeting control (siCon; all 100 nM), and mRNA expression levels (mean ± S.D., n = 6) of MMP1 measured 24 h post-stimulation by qPCR, relative to siCon treated cells, normalized to 18S rRNA. Statistical comparisons are versus IL-1 + OSM + siCon (Student’s two-tailed unpaired t test), where ***, p<0.001; **, p <0.01; *, p<0.05; ns = not significant. The data are pooled from three separate experiments, each using chondrocyte cultures from different donors.

Given the discord between the transient increase of cFos by IL-1+OSM [9, 10, 25] and the requirement for new protein synthesis at times after cFos expression in terms of *MMP1* expression, these findings strongly indicated the requirement of additional transcriptional regulator(s) as shown for the expression of *MMP13* [10]. Employing a GEO-genome-wide analysis of IL-1+OSM-induced genes in chondrocytes (accession file GSE86578), we further confirmed that the temporal expression profile of *MMP1* was maximal at 24 h (Fig 4A) whilst qPCR confirmed significant expression at 24h in these populations (Fig 4B). Moreover, this gene array analysis identified a number of transcriptional regulatory genes with an expression profile similar to *ATF3* which has been shown to be an important AP-1-binding factor for *MMP13* expression [10]. A siRNA screen of these regulators, including *ATF3*, to assess *MMP1* repression confirmed a requirement for both *FOS* (cFos) and *JUN* (cJun) but not *ATF3* for *MMP1* expression (Fig 4C). Notably, only silencing of *CSRNP1* selectively and significantly repressed *MMP1* with no significant inhibition of *MMP13* (S2 Fig).

### Sustained CSRNP1 expression contributes to *MMP1* transcription

**Fig 5:**
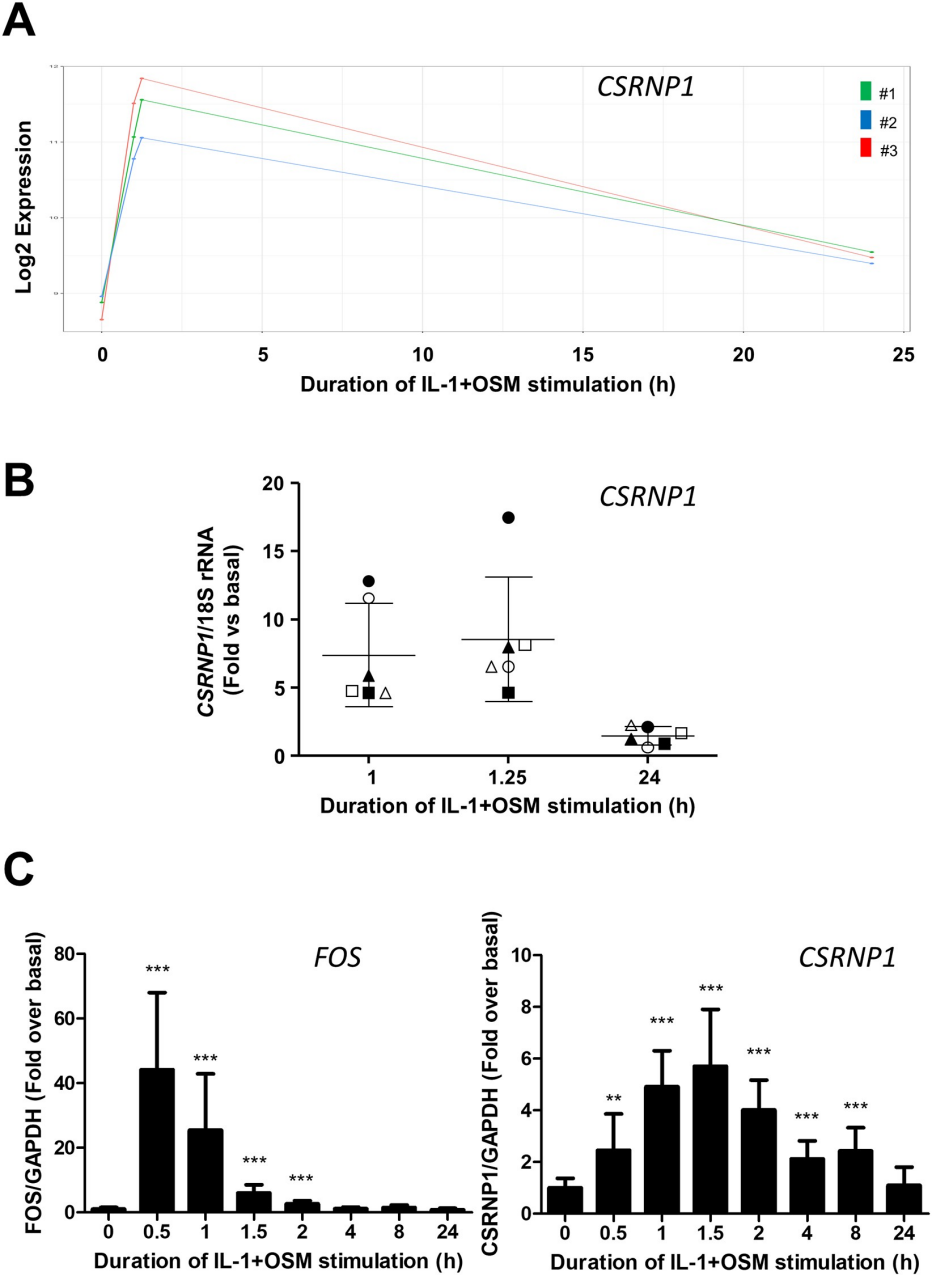
IL-1+OSM induces sustained expression of CSRNP1 mRNA. Human chondrocytes were treated with IL-1 (0.05 ng/mL) in combination with OSM (10 ng/mL) for the indicated time points and total RNA isolated. (A) RNA from three separate populations (#1, #2 and #3) was profiled using the Human HT-12v4 Expression Beadchip. The expression profile for *CSRNP1* from the microarray dataset for each chondrocyte population is shown. RNA from 6 (B) or 3 (C) separate chondrocyte populations was subjected to qPCR for *FOS* (C) or *CSRNP1* (B and C), and relative expression levels (normalized to 18S rRNA or *GAPDH* as indicated) at the indicated times determined. The plots show mean ± SD. Data from the populations used in the microarray analyses in (A) are highlighted by the solid symbols (#1, ▲; #2 ■; #3, ●). (C) Statistical comparisons are: ***, p<0.001; **, p<0.01; *, p<0.05 versus basal.

**Fig 6:**
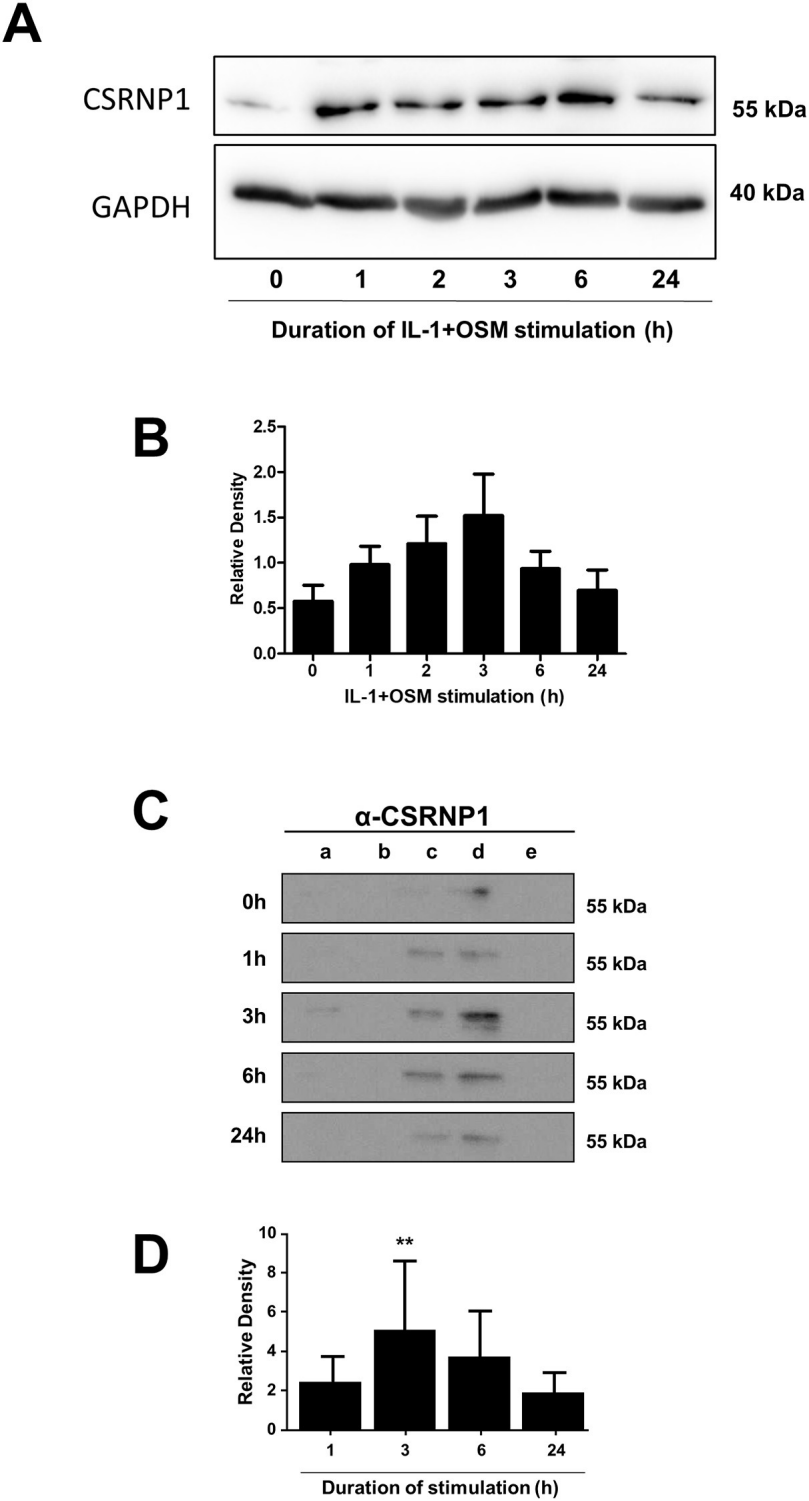
IL-1+OSM induces nuclear expression of CSRNP1. Human chondrocytes were stimulated with IL-1 (0.05 ng/mL) in combination with OSM (10 ng/mL) for the indicated durations. Protein from either whole cell lysates (A) or subcellular fractions (C: a, cytosolic; b, membrane-bound; c, soluble nuclear; d, chromatin-bound; e, cytoskeletal) were resolved using SDS-PAGE and immunoblotted with an antibody to CSRNP1. Combined densitometric scans of separate blots for CSRNP1 in cell lysates (n = 3; B) or pooled soluble and chromatin-bound nuclear fractions (n = 5; D), relative to t = 0 h, are shown and were obtained from separate chondrocyte populations. All data are presented as mean (± SD), where **, p<0.01, IL-1+OSM-treated compared to control; ANOVA.

The siRNA data above implicated a selective role for CSRNP1 in contributing to the transcriptional activation of *MMP1* (but not *MMP13*). Furthermore, expression profiling also revealed that *CSRNP1* expression was evident by 1 h and peaked at 1.25 h (Fig 5A and 5B). In order to correlate *CSRNP1* expression with *FOS*, a detailed time-course was performed which showed peak *CSRNP1* expression to follow maximal *FOS* expression (Fig 5C). Importantly, CSRNP1 protein was present from 1 to 24 h (Fig 6A–6C) and appeared to be exclusively nuclear (both soluble and chromatin-bound fractions) following subcellular fractionation (Fig 6C), peaking at 3 h (Fig 6B and 6D).

**Fig 7:**
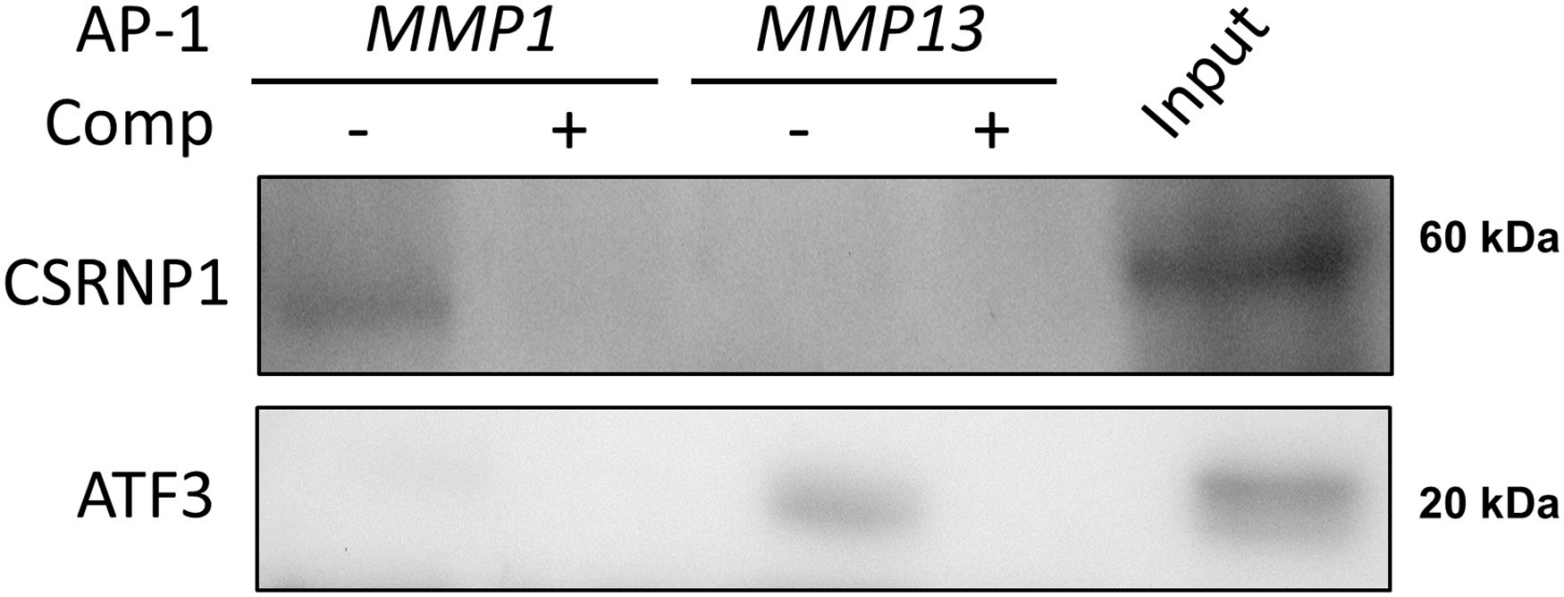
CSRNP1 selectively binds the proximal *MMP1* AP-1 motif at a transcriptionally active time-point in IL-1+OSM-stimulated chondrocytes. Human chondrocytes were treated with IL-1 (0.2 ng/mL) in combination with OSM (10 ng/mL) for 3 h. Nuclear lysates were prepared and subjected to either Western blotting alone (input) or DAPA with CSRNP1 or ATF3 antibodies, as described in the Materials and Methods. Non-biotinylated AP-1 oligonucleotides (50x excess; Comp) were used to confirm specificity of binding in the DAPAs. The data are representative of 3–5 separate experiments each using chondrocyte cultures from different donors.

*In silico* analyses using the ENCODE database [34] indicated that a consensus-like CSRNP1 motif AGAGTN [33] was present within the proximal ChIPSeq AP-1 (cJun) binding site within the *MMP1* promoter, with a 1 bp mismatch. We therefore performed DAPA analyses which, importantly, confirmed specific CSRNP1 binding to this sequence, compared to the equivalent motif within *MMP13* (Fig 7), thus further confirming the selective specificity of CSRNP1 for *MMP1*. Finally, we also assessed the ability of the *MMP1/13* AP-1 sequences to bind ATF3 and only observed binding to the *MMP13* AP-1 *cis* element (Fig 7) in line with previous findings [10].

## Discussion

Recent evidence indicates that IL-1+OSM-induces *MMP13* expression via transcriptional modulation by initial AP-1 (cFos) expression and then by subsequent ATF3 transcriptional activity [10]. In this context, the transcriptional regulation of *MMP1*, which is also potently induced by this stimulus [6–9, 35–37], has remained undefined. Although MMP-13 is the most potent collagenolytic proteinase against type II collagen [38], the abundance of MMP-1 compared to MMP-13 following pro-inflammatory stimuli [35, 37, 39] also results in significant cleavage of type II collagen resulting in irreversible cartilage damage [40]. Since *MMP1* and *MMP13* appear to be differentially regulated following IL-1+OSM stimulation [8, 9, 26], in the study presented herein we sought to identify transcriptional modulators that are selective for *MMP1* in comparison to our previous observations for *MMP13* [10].

Employing ChIP assays with cFos, phosphorylated RNA Pol II and acetylated histone H3 antibodies, we demonstrated that cFos transcriptional control was similarly important for regulating *MMP1* expression as observed for *MMP13* [10]. cFOS was recruited to the MMP1 promoter 1 hour post-stimulation, however pRNA pol II was not detected at this timepoint, rather being recruited to the promoter 24 hours post-stimulation. These data indicate that despite cFOS recruitment to the *MMP1* promoter, *MMP1* transcription does not occur at this early time point. Furthermore, the inhibition of protein synthesis after IL-1+OSM stimulation demonstrates that when protein synthesis is inhibited within the first 4 hours of stimulation, no *MMP1* expression is observed 24 hours post-stimulation. However if protein synthesis is not inhibited until after 6 hours of IL-1+OSM, *MMP1* expression is observed following 24 hour stimulation. The requirement for the *de novo* synthesis of transcriptional regulators following cell activation has been previously reported in the literature [10, 41, 42]. Taken together, these data strongly suggest that *MMP1* expression is dependent on *de novo* protein synthesis of additional factors post-*FOS* but prior to *MMP1* expression.

In order to identify such factors, GEO-genome-wide microarray analyses with subsequent siRNA assays were performed. These studies identified several transcriptional regulators which had an elevated expression level 1–1.25 hours post IL-1+OSM stimulation. In comparing the effect of siRNA knock down of these transcriptional regulators on *MMP* expression [10], only *CSRNP1* was found to be selective and specific for the regulation of *MMP1* expression over MMP13. This study indicated that *MMP1* expression was indeed dependent on cFos but that other *FOS* family members also affected *MMP1* expression via promoting AP-1-dependent gene expression. In this respect, *FOSL1* and *FOSB* were induced (see S3 Fig) and gene silencing of both resulted in significant *MMP1* repression. These analyses are also in agreement with other studies indicating a role for cytokine induction of the NFκB component RelA (p65) for *MMP1* expression in chondrocytes [15, 43–45]. We show here that siRNA silencing of *RELA* leads to a significant decrease in *MMP1* expression although to a much lesser extent than seen for *FOS* or *FOSL1*.

Little is known about the role of CSRNP1 as a transcriptional regulator. Here we provide the first evidence that CSRNP1 has an important modulatory role in affecting cytokine-induced *MMP1* expression in human articular chondrocytes. The consensus sequence for CSRNP1 binding is an AP-1-like binding motif 5’-AGAGTN-3’ [33] and both *MMP1* and *MMP13* have similar AP-1-like binding motifs within their proximal promoter regions. The *MMP1* sequence is 5’-TGAGTCA-3’ and the *MMP13* sequence has a C instead of a G (underlined) resulting in 5’-TGACTCA-3’. This G to C switch indicates preferential binding of CSRNP1 to the *MMP1* compared to the *MMP13* AP-1-like *cis* element as observed via DAPA analyses. This may be indicative that the proximal *MMP1* AP-1-like sequence is a specific *cis* element capable of binding CSRNP1 in chondrocytes. Furthermore, *in silico* analysis indicates the presence of other putative CSRNP1 binding sites located within the *MMP1* proximal promoter, but not within *MMP13*, that may also contribute to the expression of *MMP1*. Since no evidence for reproducible ATF3 binding to the *MMP1* AP-1 *cis* element was observed employing DAPAs in contrast to *MMP13*, and gene silencing of *ATF3* resulted in no significant decrease in *MMP1* expression, *MMP1* appears unlikely to be an ATF3-responsive gene. Thus, CSRNP1 and ATF3 may respectively have mutually exclusive roles in the cytokine-mediated expression of *MMP1* and *MMP13* in chondrocytes.

In conclusion, our data presented here indicate that a potent catabolic stimulus such as IL-1+OSM induces the expression of specific transcriptional regulators that contribute to *MMP1* gene activation which may well reflect the markedly enhanced levels of expression that are observed compared to either cytokine alone [35, 39]. Since multiple pro-inflammatory mediators are likely to be prevalent in most inflammatory situations, this study further highlights the complexity of *MMP* gene regulation that occurs in human chondrocytes when subjected to inflammatory cues.

## Supporting information

S1 Fig Confirmation of antibody specificity using human chondrocyte lysates. The specificity of each antibody used in the study was confirmed using nuclear lysates prepared as described in the Methods from primary human articular chondrocytes either unstimulated or stimulated with IL-1 (0.2 ng/mL) in combination with OSM (10 ng/mL). Following SDS-PAGE, proteins were transferred to PVDF membranes and probed with the indicated antibodies. Full-length blots are presented to highlight the specific immuno-reactivity of each antibody with an arrow indicating the expected molecular mass.
(TIF)

S2 Fig Multiple transcriptional regulators impact on MMP13 gene expression. Following transfection with siRNA specific for the indicated genes or non-targeting control (siCon; all 100 nM), Human chondrocytes were treated with IL-1 (0.05 ng/ml) in combination with OSM (10 ng/ml) for 24 hours. RNA isolated from three separate populations were subjected to qPCR, relative to siCon control normalised to 18S rRNA. Statistical comparisons are: ***, p<0.001; **, p<0.01; *, p<0.05; ns = not significant, versus siCon. The effect of silencing ATF3, FOS, FOSB, FOSL1, IER3, IRF1, JUN and RELA on *MMP13* expression was originally published in the Journal of Biological Chemistry. Chan *et al*. (2017)[10], Copyright the American Society for Biochemistry and Molecular Biology.
(TIF)

S3 Fig Multiple transcriptional regulators are induced following IL-1+OSM stimulation of human chondrocytes. Human chondrocytes were treated with IL-1 (0.05 ng/mL) in combination with OSM (10 ng/mL) for 1, 1.25 or 24 h and total RNA isolated. RNA from three separate populations (#1, #2 and #3 as presented in Figs 4 and 5) was profiled using the Human HT-12v4 Expression Beadchip. The *MMP1* expression profile reported in Fig 4 is presented here for comparison with the profiles of the indicated genes from the microarray dataset for each chondrocyte population.
(TIF)
