# Supplementary figures and images for: Cytokine-induced cysteine- serine-rich nuclear protein-1 (CSRNP1) selectively contributes to MMP1 expression in human chondrocytes
Source: PLoS One. 2018 Nov 15;13(11):e0207240. doi: 10.1371/journal.pone.0207240 (PMC6237337; doi:10.1371/journal.pone.0207240)

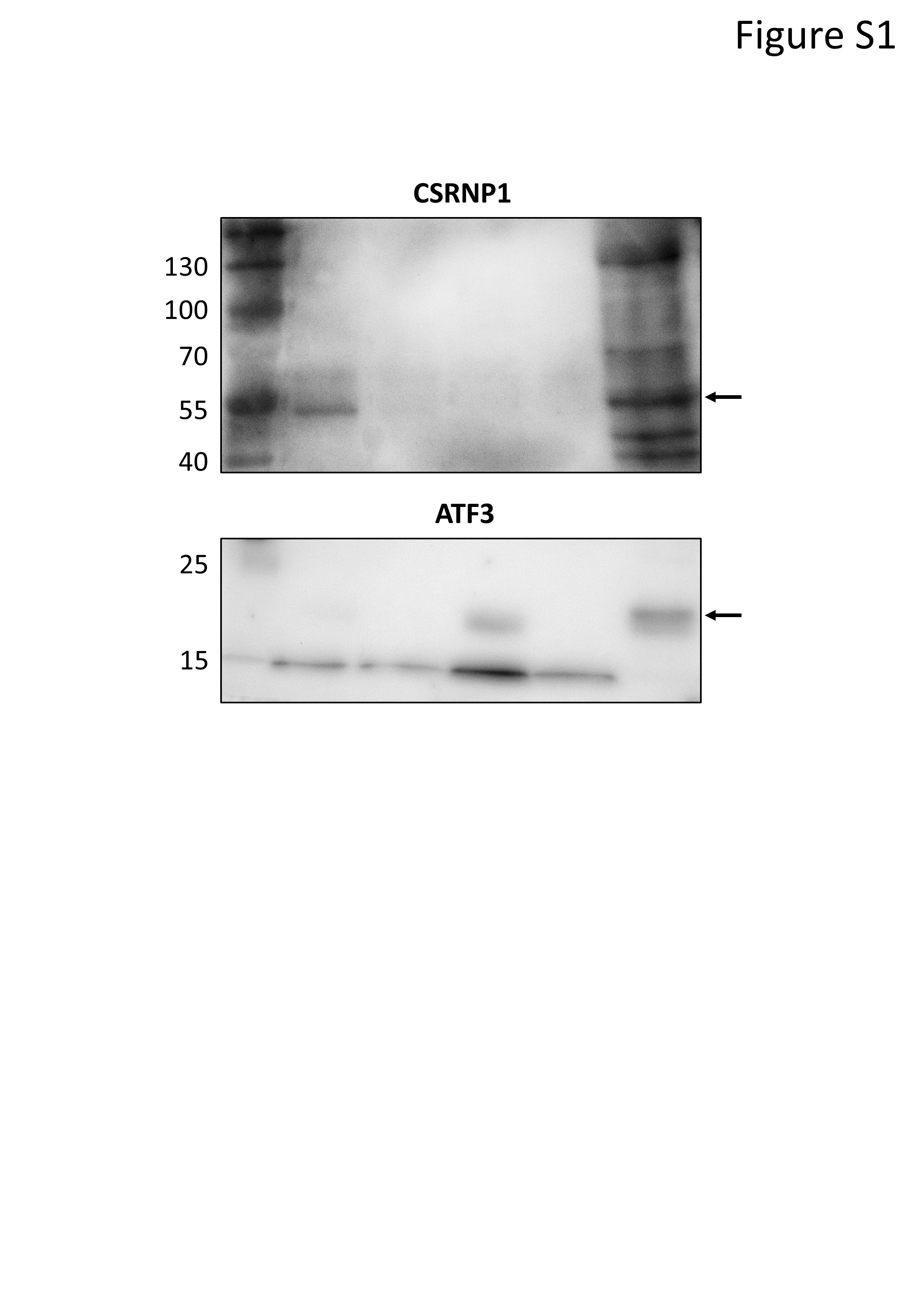

Supplement: S1 Fig — The specificity of each antibody used in the study was confirmed using nuclear lysates prepared as described in the Methods from primary human articular chondrocytes either unstimulated or stimulated with IL-1 (0.2 ng/mL) in combination with OSM (10 ng/mL). Following SDS-PAGE, proteins were transferred to PVDF membranes and probed with the indicated antibodies. Full-length blots are presented to highlight the specific immuno-reactivity of each antibody with an arrow indicating the expected molecular mass. (TIF) [file pone.0207240.s001.tif]

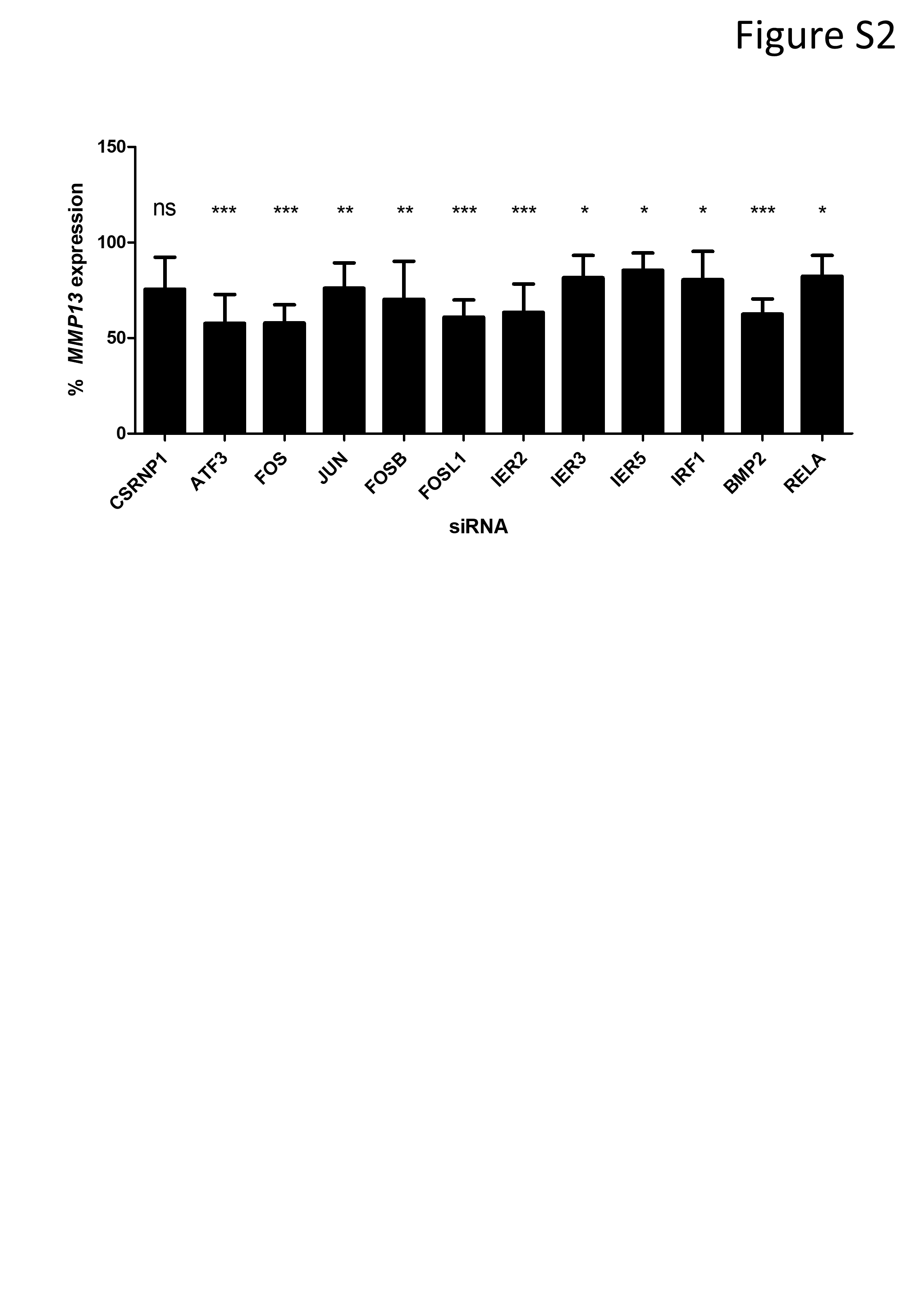

Supplement: S2 Fig — Following transfection with siRNA specific for the indicated genes or non-targeting control (siCon; all 100 nM), Human chondrocytes were treated with IL-1 (0.05 ng/ml) in combination with OSM (10 ng/ml) for 24 hours. RNA isolated from three separate populations were subjected to qPCR, relative to siCon control normalised to 18S rRNA. Statistical comparisons are: ***, p<0.001; **, p<0.01; *, p<0.05; ns = not significant, versus siCon. The effect of silencing ATF3, FOS, FOSB, FOSL1, IER3, IRF1, JUN and RELA on MMP13 expression was originally published in the Journal of Biological Chemistry. Chan et al. (2017)[10], Copyright the American Society for Biochemistry and Molecular Biology. (TIF) [file pone.0207240.s002.tif]

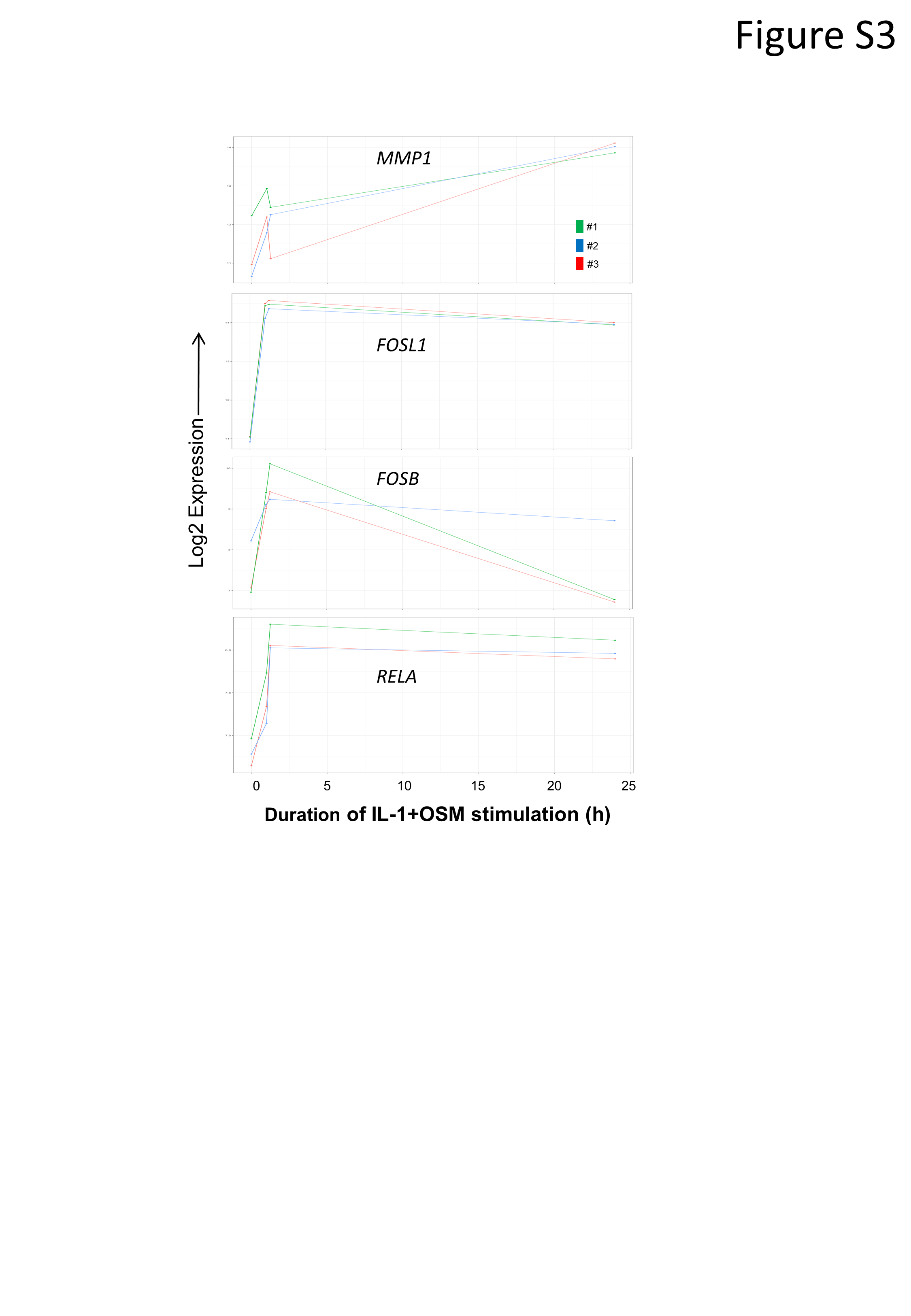

Supplement: S3 Fig — Human chondrocytes were treated with IL-1 (0.05 ng/mL) in combination with OSM (10 ng/mL) for 1, 1.25 or 24 h and total RNA isolated. RNA from three separate populations (#1, #2 and #3 as presented in Figs 4 and 5) was profiled using the Human HT-12v4 Expression Beadchip. The MMP1 expression profile reported in Fig 4 is presented here for comparison with the profiles of the indicated genes from the microarray dataset for each chondrocyte population. (TIF) [file pone.0207240.s003.tif]
